# Deficiency of Smad7 Enhances Cardiac Remodeling Induced by Angiotensin II Infusion in a Mouse Model of Hypertension

**DOI:** 10.1371/journal.pone.0070195

**Published:** 2013-07-23

**Authors:** Li Hua Wei, Xiao Ru Huang, Yang Zhang, You Qi Li, Hai-yong Chen, Rainer Heuchel, Bryan P. Yan, Cheuk-Man Yu, Hui Yao Lan

**Affiliations:** 1 Department of Medicine and Therapeutics, Prince of Wales Hospital, The Chinese University of Hong Kong, Hong Kong, China; 2 Li Ka Shing Institute of Health Sciences, The Chinese University of Hong Kong, Hong Kong, China; 3 Department of Clinical Science, Intervention and Technology, Karolinska Institutet, Stockholm, Sweden; Osaka University Graduate School of Medicine, Japan

## Abstract

Smad7 has been shown to negatively regulate fibrosis and inflammation, but its role in angiotensin II (Ang II)-induced hypertensive cardiac remodeling remains unknown. Therefore, the present study investigated the role of Smad7 in hypertensive cardiopathy induced by angiotensin II infusion. Hypertensive cardiac disease was induced in Smad7 gene knockout (KO) and wild-type (WT) mice by subcutaneous infusion of Ang II (1.46 mg/kg/day) for 28 days. Although equal levels of high blood pressure were developed in both Smad7 KO and WT mice, Smad7 KO mice developed more severe cardiac injury as demonstrated by impairing cardiac function including a significant increase in left ventricular (LV) mass (P<0.01),reduction of LV ejection fraction(P<0.001) and fractional shortening(P<0.001). Real-time PCR, Western blot and immunohistochemistry detected that deletion of Smad7 significantly enhanced Ang II-induced cardiac fibrosis and inflammation, including upregulation of collagen I, α-SMA, interleukin-1β, TNF-α, and infiltration of CD3^+^ T cells and F4/80^+^ macrophages. Further studies revealed that enhanced activation of the Sp1-TGFβ/Smad3-NF-κB pathways and downregulation of miR-29 were mechanisms though which deletion of Smad7 promoted Ang II-mediated cardiac remodeling. In conclusions, Smad7 plays a protective role in AngII-mediated cardiac remodeling via mechanisms involving the Sp1-TGF-β/Smad3-NF.κB-miR-29 regulatory network.

## Introduction

Hypertensive heart disease is a major global health problem and a considerable cause of cardiovascular morbidity and mortality [1]. Fibrosis and inflammation are two key pathologic features in hypertensive cardiac remodeling which causes left ventricular (LV) hypertrophy and can lead to LV dysfunction and heart failure [2]–[4]. Angiotensin II (Ang II) is a well-established mediator of the renin-angiotensin system (RAS) in the pathogenesis of hypertensive heart disease [3]–[5]. In addition to its hypertensive effect, Ang II may induce cardiac remodeling by promoting cardiac hypertrophy, inflammation, and fibrosis via a number of signaling pathway [3]–[5]. It has been reported that nuclear factor κB (NF-κB) pathway plays an important role in Ang II-induced cardiac inflammation [6]. Recent studies showed that Ang II mediates cardiovascular fibrosis via transforming growth factor-β (TGF-β)-dependent and independent Smad signaling [7], [8]. It is now well accepted that upon binding to its receptor, TGF-β1 activates two downstream proteins, Smad2 and Smad3, to exert its biological effects, which are negatively regulated by Smad7, a downstream inhibitory Smad in TGF-β signaling [9]. In the context of fibrosis, we and other investigators have found that both TGF-β1 and Ang II can act by stimulating Smad3, not Smad2, to mediate fibrosis in vitro and in a number of diseases including obstructive nephropathy, Ang II-induced hypertensive nephropathy and cardiopathy, ischemic cardiac remodeling [10]–[15], which is inhibited by Smad7 under a variety of pathological conditions[14]–[18]. In addition, we have also identified that Smad7 plays a key role in negatively regulating renal inflammation by blocking activation of NF-κB signaling pathway [18]–[20]. Thus, Smad7 is an important inhibitor of both TGF-β/Smad and NF-κB signaling pathways and plays a negatively regulating role in renal fibrosis and inflammation. However, the role of Smad7 in hypertensive cardiac remodeling remains unclear. Therefore, in the present study, by using Smad7 KO mice, we investigated the functional role of Smad7 in Ang II-induced hypertensive cardiac disease.

## Materials and Methods

### Mouse Model of Ang II–induced Hypertension

Smad7 KO and their littermate WT mice CD-1 background, male, aged 8–10 weeks, 30–35 g) were used in this study. The generation of the Smad7 KO mice by functionally deleting Smad7 gene exon I has been described previously and the genotype was confirmed by PCR [21]. Hypertensive cardiac remodeling was induced by subcutaneous infusion of Ang II at a dose of 1.46 mg/kg per day for 28 days via osmotic minipumps (Model2004, ALZA Corp, Palo Alto, CA) as described previously [11], [12]. Control mice received saline instead of AngII. Mice were euthanized at day 28 after Ang II infusion. Systolic blood pressure was measured in conscious and calm mouse by the non-invasive tail-cuff method using the CODA blood pressure system (Kent Scientific, Torrington, CT) according to the manufacturer’s instruction. LV tissues were collected for immunohistochemistry, real-time PCR, and western blot analysis. The experimental procedures were approved by the Animal Ethics Committee of The Chinese University of Hong Kong (Permit No. 1165-05).

### Echocardiography

Transthoracic echocardiography was performed in both Smad7 KO and WT mice before and at day 28 after Ang II infusion. Echocardiography was conducted using a Vevo770 high resolution ultrasound imaging system (VisualSonics Inc., Toronto, Canada) with a RMV 707B scanhead (30 MHz) (VisualSonics Inc., Toronto, Canada). The LV ejection fraction (LVEF =  [(LVDD^3^−LVSD^3^)/LVDD^3^]×100%), fractional shortening (FS = [(LVDD−LVSD)/LVDD]×100%) and LV mass (mg) = 1.055×[(IVS+LVDD+ LVPW)^3^−(LVDD)^3^] were calculated.

### Immunohistochemistry

Immunohistochemistry was performed in paraffin sections using a microwave-based antigen retrieval method [22]. The antibodies used in this study included: collagen I (Southern Biotech Inc., Birmingham, AL), α-SMA(R&D, Minneapolis, MN), TNFα and IL-1β (Santa Cruz Biotechnology, Santa Cruz, CA), CD3+T cells (Abcam, Cambridge, UK), macrophages (F4/80+) (Serotec, Oxford, UK). All slides (except sections stained with antibodies against α-SMA) were counterstained with haematoxylin. The percentage of positive staining for collagen I, α-SMA, TNFα, IL-1β was measured by a quantitative image-analysis system (Image-Pro Plus 6.5, Media Cybernetics, Silver Spring, MD) in 5 consecutive fields of LV tissues under a ×20 power field of microscope, while positive cells per mm^2^ for CD3 and F4/80 were counted under the ×20 power field of microscope in 5 random areas of LV tissues using a 0.25 mm^2^ graticule fitted in the eyepiece of the microscope as previously described [12].

### Real-time PCR

The RNA was extracted LV tissues and real-time PCR analysis was performed by Bio-Rad iQ SYBR Green supermix with the Opticon2 (Bio-Rad, Hercules, CA, USA) using primers of collagen I, α-SMA, IL-1β, TNFα, and GAPDH as described previously [12], [18]. Reaction specificity was confirmed by melting curve analysis. The ratio against housekeeping gene GAPDH for individual mRNA was calculated and expressed as mean±standard errors (SE). In addition, miR-29b expression was detected by real-time PCR using the Taqman microRNA Assay (Applied Biosystems, Foster City, CA) with small nuclear RNA U6 as an endogenous control for normalization as previously described [23].

### Western Blot Analysis

The protein from LV tissues was extracted for Western blot analysis as described previously [12], [18]. Briefly, after blocking nonspecific binding with 5% BSA, membranes were incubated overnight at 4°C with primary antibodies against phospho-p65 (ser276), phospho-IκBα(ser32) and IκBα (Cell Signaling), p65, phospho-Smad2/3, Smad2/3, Smad7 and Sp1(Santa Cruz), collagen I (Southern Biotech), α-SMA(DAKO, Carpinteria, CA), GAPDH (Chemicon, Temecula, CA) were used. After being washed, the membranes were then incubated with IRDye 800 conjugated secondary antibodies (Rockland Immunochemicals, Gilbertsville, PA, USA). The signals were detected with an Odyssey Infrared Imaging System (Li-COR Biosciences, Lincoln, NE, USA) and quantified with Image J (National Institutes of Health, Bethesda, MD, USA). The ratio for the protein examined was normalized against GAPDH.

### Statistical Analyses

Data obtained from this study were expressed as mean±SE. Statistical analyses were performed using one-way ANOVA followed by Newman-Keuls multiple comparison test from GraphPad Prism 5.0 (Graph Pad Software, San Diego, CA).

## Results

### Smad7 Deficiency Aggravates Ang II–induced Cardiac Dysfunction

**Figure 1:**
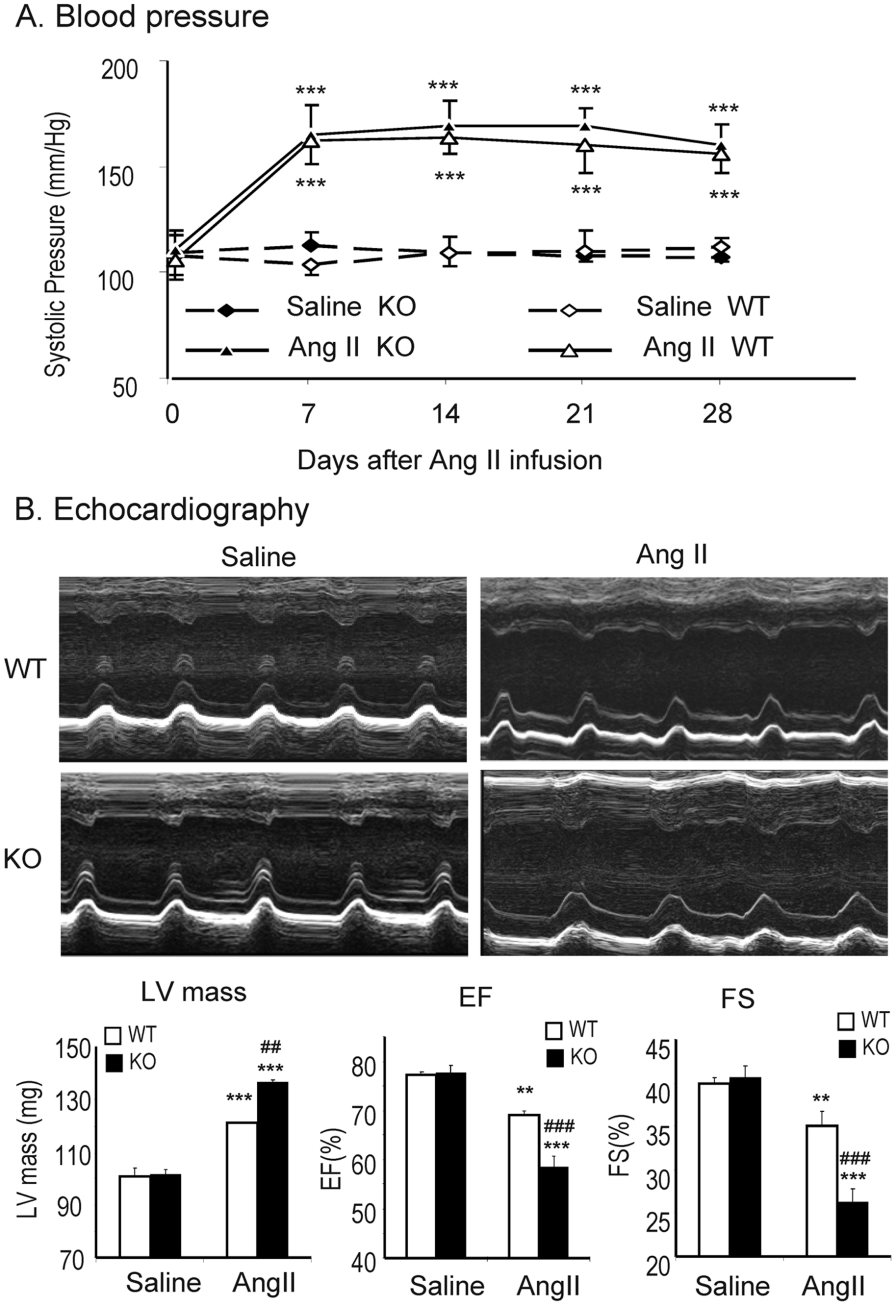
Mice lacking Smad7 develop more severe cardiac dysfunction. (A) Effect of Smad7 deletion on the systolic blood pressure in response to Ang II infusion. (B) Representative echocardiography at day 28 after saline or Ang II infusion in Smad7 WT and KO mice. Results show that Smad7 KO mice significantly increase LV mass, reduce LVEF and FS when compared with Smad7 WT mice after Ang II infusion. Data represent the mean±SE for a group of 6 mice. **P<0.01, ***P<0.001 vs saline control; ^##^P<0.01,^ ###^P<0.001 vs Ang II–infused Smad7 WT mice.

We first determined the functional role of Smad7 in hypertensive cardiac disease induced by Ang II in Smad7 KO mice. Although Ang II infusion caused equal high levels of blood pressure in both Smad7 KO and WT mice (Fig. 1A),Smad7 KO mice developed more severe cardiac injury including a significant increase in LV mass and reduction in LV ejection fraction (EF) and fractional shortening (FS) when compared to WT mice (Fig. 1B). However, changes in LVDd, LVDs, LVPWd, and LCPWs were not reached statistical significance between Smad7 KO and WT mice after Ang II infusion (data not shown).

### Disruption of Smad7 Exacerbates Ang II-induced Cardiac Fibrosis and Inflammation

**Figure 2:**
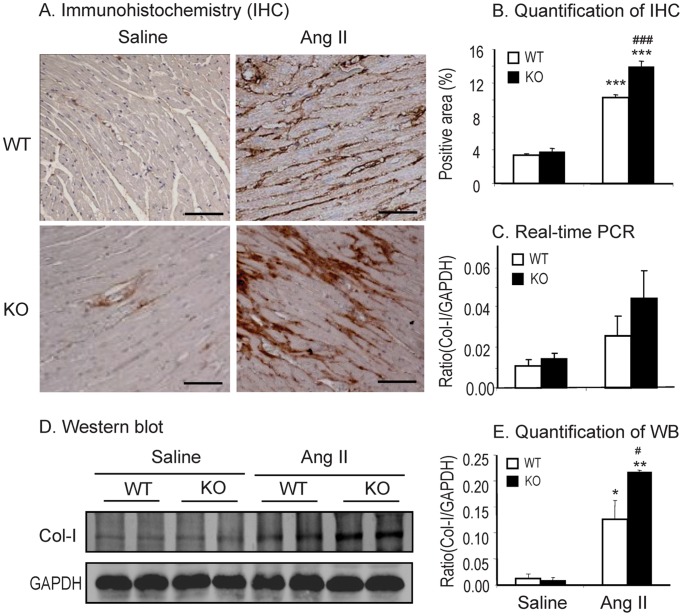
Loss of Smad7 promotes collagen I expression during cardiac fibrosis in response to Ang II infusion. (A and B) Immunohistochemical staining and quantitative analysis of cardiac collagen I accumulation. (C) Real-time PCR analysis of collagen I (Col-I) mRNA expression. (D and E ) Western blot analysis of collagen I expression. Data represent the mean±SE for a group of 6 mice. *P<0.05, **P<0.01, ***P<0.001 vs saline control; ^#^P<0.05, ^###^P<0.001 vs Ang II–infused Smad7 WT mice. Scale bar = 100 µm.

**Figure 3:**
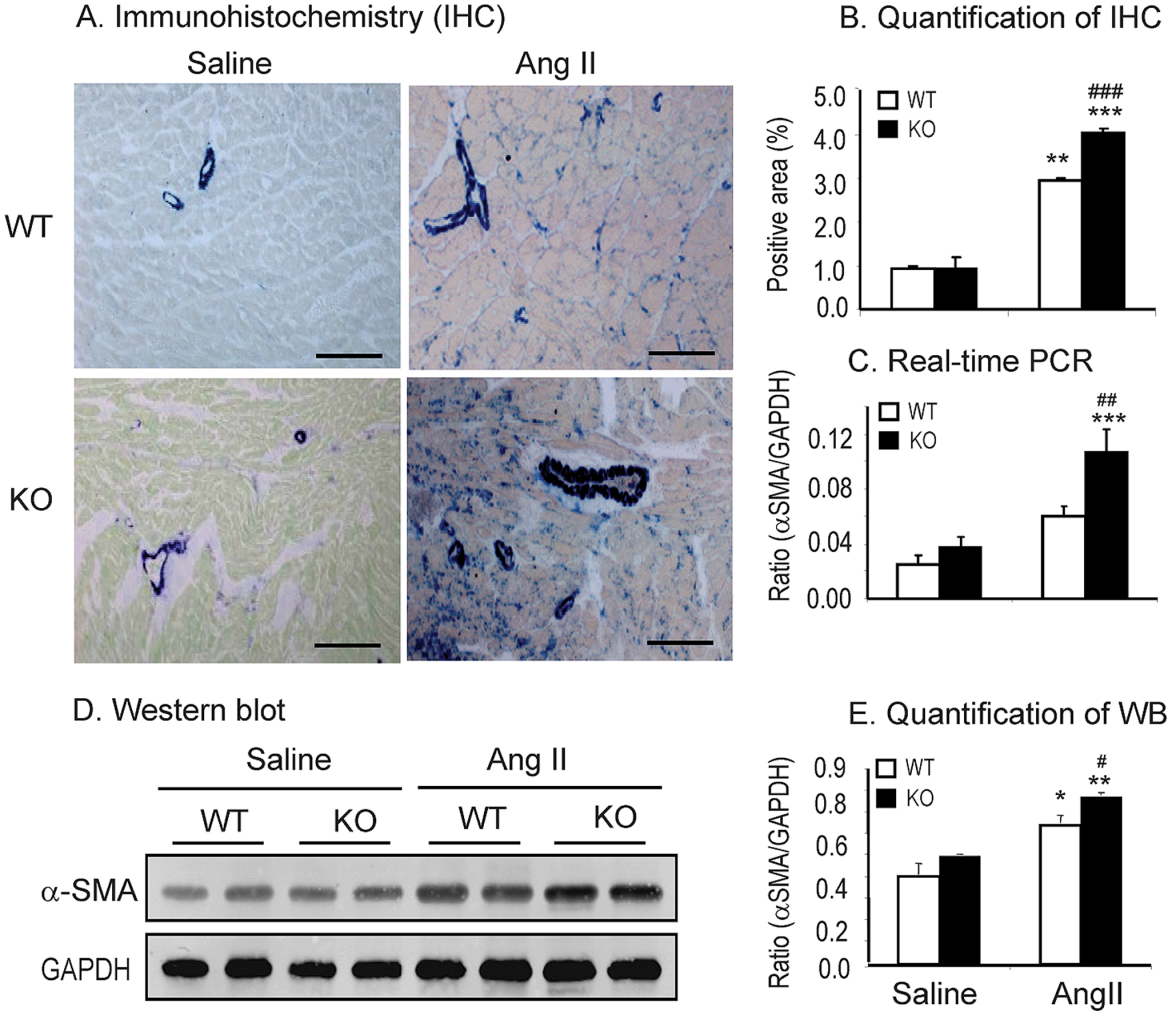
Loss of Smad7 promotes α-SMA expression during cardiac fibrosis in response to Ang II infusion. (A and B) Immunohistochemical staining and quantitative analysis of cardiac α-SMA accumulation. (C) Real-time PCR analysis of α-SMA mRNA expression. (D and E ) Western blot analysis of α-SMA expression. Data represent the mean±SE for a group of 6 mice. *P<0.05, **P<0.01, ***P<0.001 vs saline control; ^#^P<0.05, ^##^P<0.01, ^###^P<0.001 vs Ang II–infused Smad7 WT mice. Scale bar = 100 µm.

**Figure 4:**
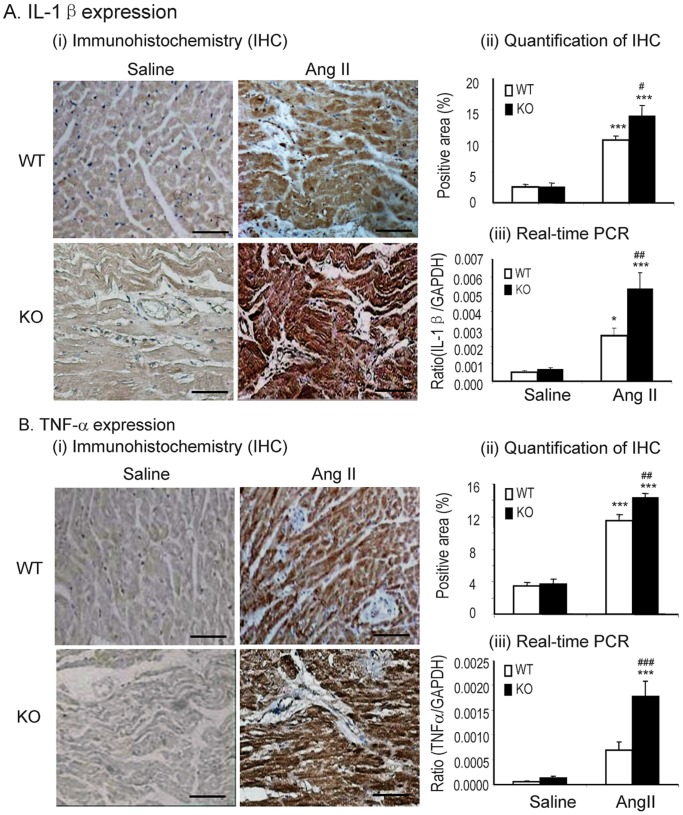
Loss of Smad7 aggravates proinflammation expression during cardiac inflammation in response to Ang II infusion. (A) IL-1β expression. (B) TNF-α expression. (i) Immunohistochemistry (IHC); (ii) quantitative analysis of immunohistochemical staining (IHC); (iii) real-time PCR analysis. Data represent the mean±SE for a group of 6 mice. *P<0.05, ***P<0.001 vs saline control; ^#^P<0.05, ^##^P<0.01, ^###^P<0.001 vs Ang II–infused Smad7 WT mice. Scale bar = 100 µm.

**Figure 5:**
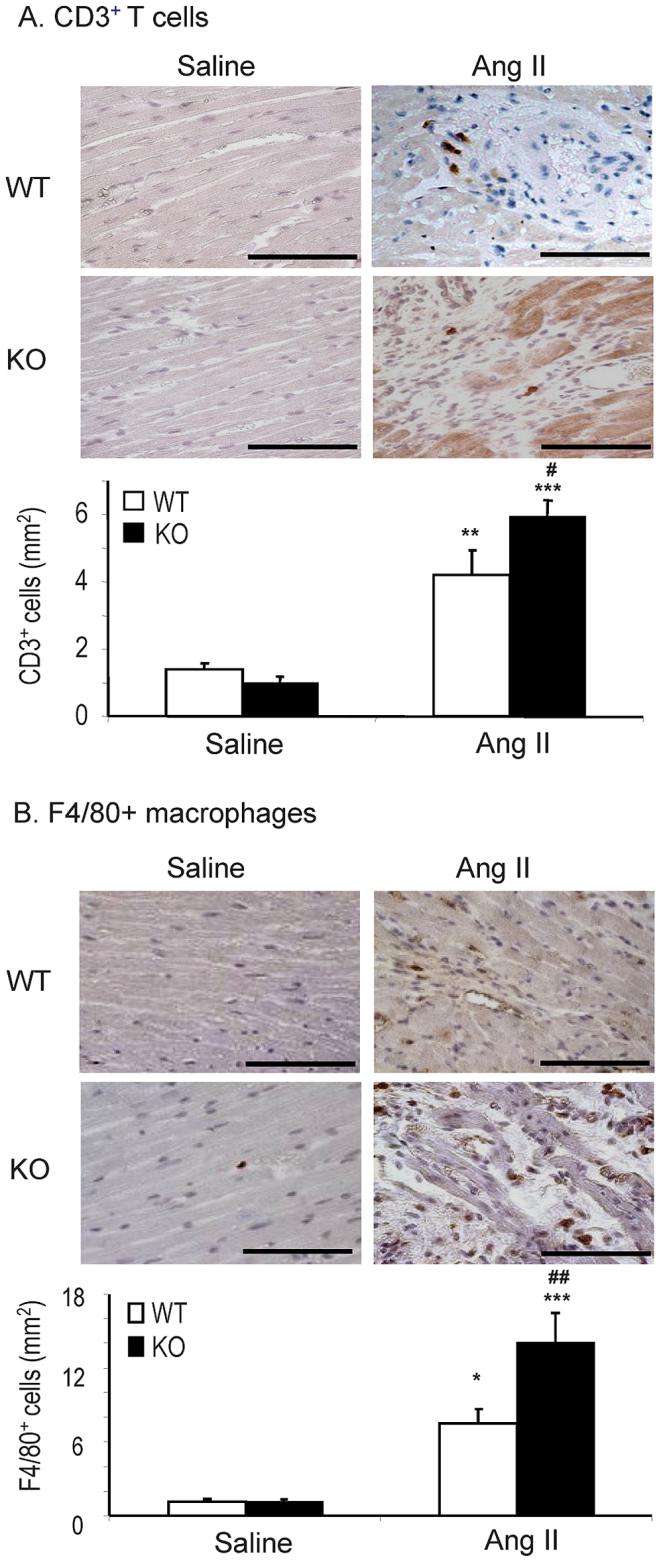
Loss of Smad7 exacerbates Ang II–induced CD3+ T cell and F4/80+ macrophage infiltration. (A) Immunohistochemical staining of CD3+ T cells. (B) Immunohistochemical staining of F4/80+ macrophages. Data represent the mean±SE for a group of 6 mice. *P<0.05, **P<0.01,***P<0.001 vs saline control; ^#^P<0.05, ^##^P<0.01 vs Ang II–infused Smad7 WT mice. Scale bar = 100 µm.

We next detected the effect of Smad7 deficiency on Ang II-induced cardiac fibrosis and inflammation. Western blot and immunohistochemistry showed that deletion of Smad7 significantly enhanced cardiac fibrosis as indicated by a marked increase in expression of α-SMA and collagen I protein (Figs. 2 and 3). These changes were also confirmed by real-time PCR at the mRNA levels with collagen 3 (Fig. 3C), although no statistical significance was reached in collagen I mRNA expression due to the variation of individual animals (Fig. 2C). Similarly, cardiac inflammation was also exacerbated in Smad7 KO mice as demonstrated by a marked upregulation of pro-inflammatory cytokines IL-1β and TNF-α (Fig. 4), and a larger number of F4/80+ macrophages and CD3+ T cells within the cardiac tissues after Ang II infusion (Fig. 5).

### Enhanced Activation of Sp1, TGF-β/Smad, and NF-κB Signaling Pathways and Downregulation of miR-29 are Mechanisms by which Deletion of Smad7 Promotes Cardiac Fibrosis and Inflammation

**Figure 6:**
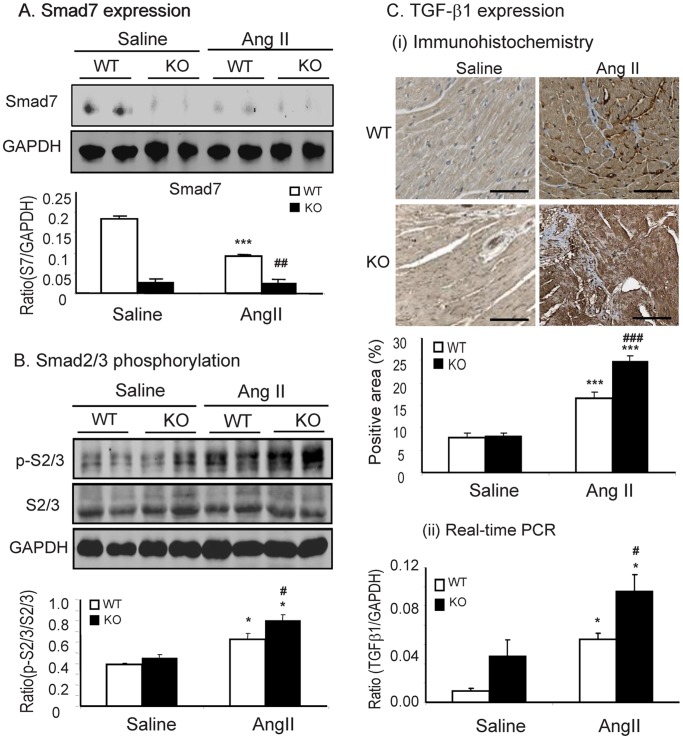
Disruption of Smad7 enhances Ang II-induced activation of TGF-β/Smad signaling during cardiac fibrosis. (A) Western blot analysis of Smad7. (B) Western blot analysis of phosphorylated Smad2/3. (C) Immunohistochemical and real-time PCR analysis of TGF-β1 expression. Results show that deletion of Smad7 enhances TGFβ1 expression and Smad2/3 signaling in response to Ang II. Data represent the mean±SE for a group of 6 mice. *P<0.05, ***P<0.001vs saline control; ^#^P<0.05,^##^P<0.01, ^###^P<0.001 vs Smad7 WT mice. Scale bar = 100 µm.

**Figure 7:**
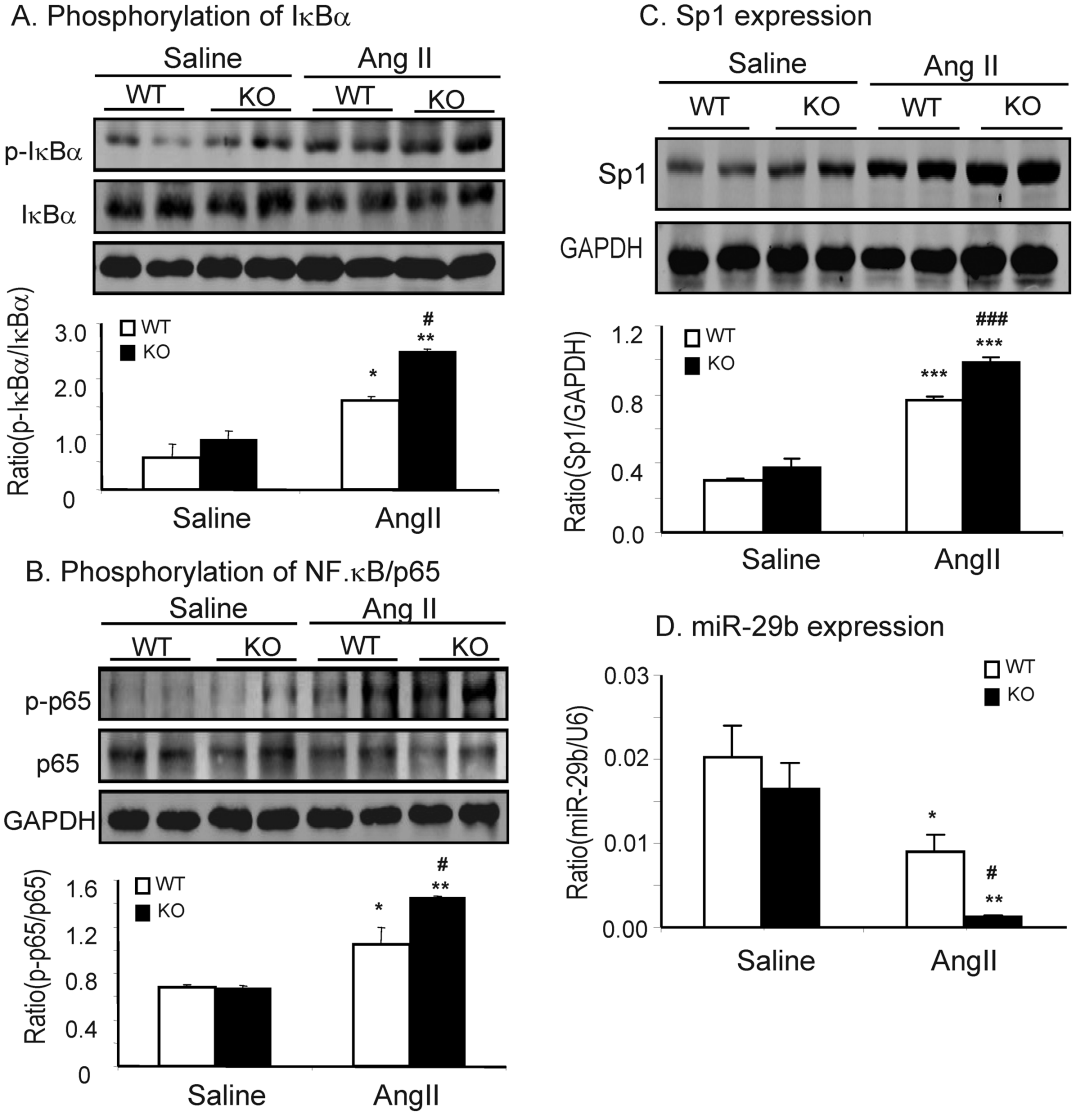
Smad7 deficiency enhances Ang II-induced NF-κB signaling activation, upregulation of Sp1, but loss of miR-29b. (A) Western blot analysis of phosphorylated IκBα. (B) Western blot analysis of phosphorylated NF-κB/p65. (C) Western blot analysis of Sp1. (D) Real-time PCR analysis of miR-29b expression. Results show that deletion of Smad7 enhances activation of NF-κB signaling by promoting phosphorylation of IκBα and p65, promotes Sp1 expression, but induces loss of cardiac miR-29b. Data represent the mean±SE for a group of 6 mice. *P<0.05, **P<0.01, ***P<0.001vs saline control; ^#^P<0.05,^###^P<0.001 vs Smad7 WT mice.

We further examined the mechanisms by which Smad7 KO mice enhanced Ang II-mediated cardiac fibrosis and inflammation. As shown by Western blot analysis, expression of cardiac Smad7 was significantly decreased in WT mice infused with Ang II; nevertheless Smad7 was almost not detectable in both saline and Ang II-infused Smad7 KO mice (Fig. 6A). Loss of cardiac Smad7 in Ang II-infused WT mice led to a marked increase in phosphorylation of Smad2/3, which was further enhanced in Ang II-infused Smad7 KO mice (Fig. 6B). Real-time PCR and immunohistochemistry also showed that enhanced Smad2/3 signaling in Ang II-infused Smad7 KO mice was associated with a further upregulation of cardiac TGF-β1 expression (Fig. 6C), demonstrating that deletion of Smad7 enhanced TGF-β/Smad signaling in the hypertensive cardiac disease. In addition, we also found that loss of cardiac Smad7 largely enhanced activation of NF-κB signaling as demonstrated by higher levels of phosphorylated IκBα and NF-κB/p65 in Smad7 KO mice when compared with Ang II-infused Smad7 WT mice (Fig. 7A, B).

Since Sp1, a ubiquitous transcription factor, is required for Ang II–induced fibrotic and inflammatory response [24], [25]. we examined Sp1 expression in the hypertensive heart and found that Ang II infusion largely upregulated cardiac Sp1 in Smad7 WT mice, which was further increased in Smad7 KO mice (Fig. 7C). Moreover, it has been shown that loss of miR-29b is associated with cardiac fibrosis and is negatively regulated by both TGF-β/Smad3 and NF-κB-YY1 [23], [26], [27], we examined whether deletion of Smad7 causes enhanced Ang II-induced loss of cardiac miR-29b during cardiac remodeling. As shown in Figure 7D, real-time PCR showed that miR-29b was significantly decreased in the hypertensive heart of Smad7 WT mice, which became almost undetectable in Ang II-infused Smad7 KO mice.

## Discussion

Increasing evidence demonstrates that Ang II is a driving force in cardiac fibrosis, inflammation and cardiac dysfunction [1], [28]. The present study provided new evidence for a protective role of Smad7 in Ang II-induced hypertensive cardiac remodeling. We detected that loss of Smad7 enhanced Ang II-induced cardiac remodeling and cardiac dysfunction. Enhanced activation of Sp1-TGF-β/Smad-NF-κB signaling pathways and loss of cardiac miR-29 were key mechanisms by which deficiency of Smad7 promoted Ang II-mediated cardiopathy.

Loss of Smad7 was a key mechanism by which Ang II induces cardiac fibrosis. Consistent with the previous observation that decreased cardiac Smad7 contributes to cardiac fibrosis [29], [30], our current study revealed that cardiac Smad7 expression was markedly reduced in response to Ang II infusion, resulting in impaired cardiac function including an increase in LV mass, reduction of LVEF and FS. Beyond this observation, by using Smad 7 KO mice, we provided a direct evidence for a functional role of Smad7 in Ang II-induced hypertensive cardiac remodeling. We found that mice lacking Smad7 developed more severe cardiac fibrosis in response to Ang II infusion and had more severe cardiac dysfunction including a significant increase in LV mass, a fall of LVEF and FS when compared with Smad7 WT mice. Once Smad7 is lost, Ang II-induced activation of Smad3 via both TGF-β-dependent and independent pathways is enhanced [7], [8], which results in enhanced Smad3-mediated fibrosis as previously reported in vitro and in a number of mouse models induced by Ang II [8], [11], [12], [14], [15], postmyocardiac infarction [13], obstructive and diabetic nephropathy [18], [31]. The observation that the lack of Smad7 promoted Ang II-induced cardiac fibrosis and dysfunction identified a critically protective role for Smad7 in Ang II-mediated cardiac remodeling.

Loss of Smad7 may also be a mechanism whereby Ang II induces cardiac inflammation via a NF-κB-dependent mechanism. We have previously showed that Smad7 is able to block inflammatory responses by preventing NF-κB from activation [18]–[20]. It is well accepted that Ang II is capable of activating NF-κB to mediate cardiovascular inflammation [6]. We have previously detected that Smad7 is able to block NF-κB-dependent inflammation by inducing IκBα, an inhibitor of NF-κB, or preventing it from degradation in a number of experimental models and in vitro [18]–[20]. In the present study, the finding that disruption of Smad7 enhanced further NF-κB activation added new evidence that Smad7 protects against Ang II-induced cardiac inflammation through inhibition of the NF-κB pathway.

Upregulation of Sp1 pathway may also contribute to promote Ang II-mediated cardiac remodeling in Smad7 KO mice. Sp1 is required for Ang II–induced fibrotic and inflammatory response [24], [25]. It has been demonstrated that Sp1 can interact with both Smad3 and NF-κB to play a critical role in fibrosis and inflammation [32]–[34]. Thus, Ang II-induced activation of Sp1/Smad3/NF-κB pathways may cooperate in the development of cardiac fibrosis and inflammation as seen in WT mice. Deletion of Smad7 enhanced further activation of this Sp1/Smad3/NF-κB axis in response to Ang II, which may well explain enhanced cardiac fibrosis and inflammation when Smad7 is disrupted.

Interestingly, we also found that loss of miR-29 may be an additional mechanism through which disruption of Smad7 enhances Ang II-mediated cardiac fibrosis and inflammation. miR-29b is negatively regulated by both TGF-β/Smad3 and NF-κB-YY1 regulatory circuit [23], [26], [27]. Increasing evidence shows that down-regulation of miR-29 is associated with fibrosis in a number of disease models including ischemic cardiac remodeling [26], while overexpression of miR-29b is capable of inhibiting Smad3-mediated kidney and lung fibrosis [23], [35]. miR-29b exerts an anti-fibrotic function through direct targeting of the 3′UTR regions in the mRNA for collagens I, III and IV and fibrillin and elastin [26]. It is also reported that miR-29b can interact with Sp1 to form the Sp1/NFκB/HDAC/miR-29b regulatory network in myeloid leukemia [36]. All these findings suggest that Ang II-induced loss of miR-29b via TGF-β/Smad3 and NF-κB-dependent pathways may also be an additional mechanism by which deletion of Smad7 promotes Ang II-induced cardiac remodeling.

In summary, Smad7 plays a protective role in Ang II-mediated cardiac fibrosis and inflammation and cardiac dysfunction. Upregulation of the Sp1-TGF-β/Smad-NF-κB pathway and loss of miR-29b may be mechanisms by which deletion of Smad7 enhances hypertensive cardiac remodeling. These findings suggest that Smad7 may be a novel therapeutic agent for hypertensive cardiovascular disease.
